# Body Composition Analysis Methods in Adolescent Athletes: A Systematic Review

**DOI:** 10.1055/a-2751-5759

**Published:** 2026-01-16

**Authors:** Sogand Poureghbali, Tilman Engel, Areeba Raja, Dominik Sonnenburg, Frank Mayer

**Affiliations:** 1 26583 Outpatient Clinic, Center of Sports Medicine, Universität Potsdam, Potsdam, Germany

**Keywords:** Adolescent athletes, body composition, fat mass, fat-free mass, DXA, BIA

## Abstract

Body composition analysis in adolescent athletes is critical for assessing fat mass percentage and fat-free mass. However, measurement inaccuracies can compromise results. Additionally, there is a lack of reliable reference methods to evaluate the accuracy of field measurement techniques. This review evaluates the reliability and validity of methods in adolescent athletes and provides evidence-based recommendations for best practice. The search (Pubmed and Scopus) followed Preferred Reporting Items for Systematic reviews and Meta-Analyses guidelines and PICO criteria related to adolescent athletes in bioelectrical impedance analysis, dual-energy X-ray absorptiometry, air displacement plethysmography and skinfold thickness measurements. Thirty-one studies out of 4,408 records met the eligibility criteria. Estimating fat mass percentage and fat-free mass in adolescent athletes is moderately reliable and valid. Dual-energy X-ray absorptiometry is often regarded as the criterion standard particularly for validating equations in bioelectrical impedance analysis and skinfold measurements. Its assumptions regarding tissue density and confounding factors limit precision. Air displacement plethysmography and hydrostatic weighing are limited in athletes with extreme body mass or atypical fat distribution. Recent calculation formulas validated for adolescents are rare and inadequate for athletes. In summary, two- and three-compartment models reflect reduced accuracy in adolescent athletes, making four-compartment models preferable. Field methods like bioelectrical impedance analysis and skinfolds require further validation due to the lack of reliable reference methods in this specific population.

## Introduction

Body composition analysis is critical in adolescent athletes (AAs) for evaluating fat mass percentage (%FM), fat-free mass (FFM), and growth status, especially during puberty. These data are fundamental for individualizing training, optimizing performance, and monitoring health-related conditions (e.g., energy deficiency and eating disorders). 1​ 2​ ​3​ However, accurate body composition assessment in youth remains challenging due to rapid growth, sex- and maturation-related differences, and limitations of commonly used methods. 4​

Hormonal changes during adolescence significantly affect the FFM and%FM, with differences between sexes. For instance, girls generally show a steady increase in%FM, while boys often experience a temporary decrease in%FM during puberty followed by an increase. 5​ 6​ ​7​

Moreover, different sports might place distinct physiological demands on AAs, potentially influencing body composition. 8​ For example, endurance and aesthetic sports are often associated with lower%FM, whereas strength- or power-based sports may promote greater muscle mass. 1​ In sports like wrestling, where weight class placement is crucial, inaccurate assessments can result in improper classification or unrealistic body composition goals. 9​

Various methods are used to assess body composition. Laboratory-based techniques such as dual-energy X-ray absorptiometry (DXA), hydrostatic weighing (HW), and air displacement plethysmography (ADP) offer high precision but come with limitations including cost, time, and, in the case of DXA, radiation exposure. 10​ These methods can also be affected by various factors such as movement, lung volume, and body density assumptions, especially in athletes. 11​ 12​ ​13​

Field methods like bioelectrical impedance analysis (BIA) and skinfold (SF) measurements are cost-effective and easier to access. However, they are prone to greater measurement variability due to the technician skill, hydration status, and reliance on population-specific prediction equations. 14​ 15​ 16​ ​17​

While laboratory methods are often treated as the gold standard, their relevance and accuracy in AAs remain debatable. 18​ Most previous research has focused on adult athletes, leaving a gap in validated methods for AAs. Many field methods are used without full validation in this population. Therefore, this systematic review aims to identify and evaluate recent, reliable, and validated methods for accurately assessing body composition in AAs.

## Methods

### Procedure, search strategy and selection criteria

This systematic review was conducted in accordance with the Preferred Reporting Items for Systematic Reviews and Meta-Analyses (PRISMA) guidelines to ensure methodological transparency, consistency, and comprehensive reporting. Moreover, it was registered in PROSPERO (CRD420251027825). 19​ The protocol was developed and approved before the review process began, ensuring adherence to predefined objectives and eligibility criteria.

**Fig. 1:**
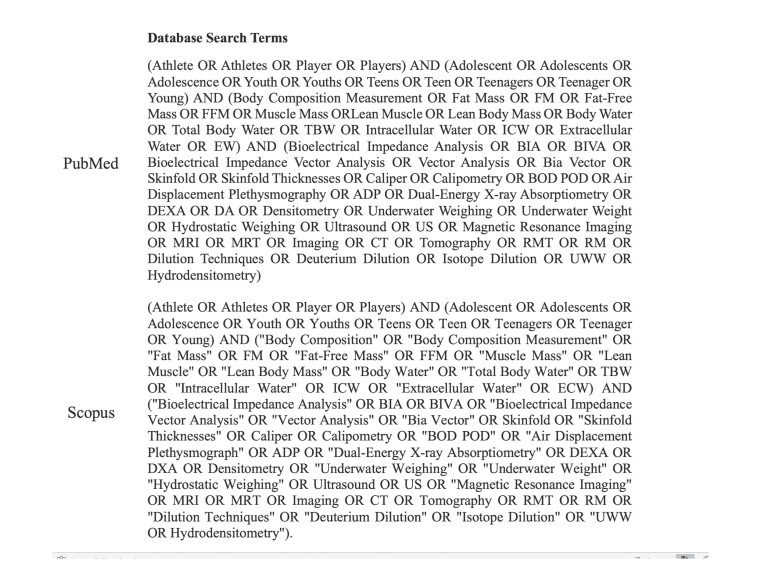
Search terms used for the literature search in PubMed and Scopus databases.

A systematic search was performed using PubMed and Scopus, restricted to studies published in English from 2000 onward. By focusing on studies published from 2000 onward, this review ensures the inclusion of more reliable, validated methods that align with current best practices in sports medicine. Older studies, while foundational, often used outdated prediction equations, different reference populations, and methodologies that may not reflect the accuracy of contemporary body composition assessment tools in AAs. 20​ The search strategy was based on the PICO framework, incorporating relevant terms and their synonyms, including athletes, adolescents, body composition measurement,%FM, FFM, muscle mass, lean body mass, total body water (TBW), BIA, SF thickness, ADP, DXA, HW, ultrasound (Ultra), magnetic resonance imaging, computed tomography, and dilution techniques (e.g., deuterium dilution). Boolean operators were applied to ensure the comprehensive retrieval of studies assessing body composition in AAs ( Fig. 1 ).

Studies were eligible if they (1) included AAs (<18 y) who were apparently healthy (excluding studies on overweight, obese, or underweight individuals), (2) assessed body composition using measurement methods rather than formula-based estimations, (3) compared or validated at least two body composition assessment methods, (4) reported outcomes related to body composition components, and (5) followed a cross-sectional or longitudinal study design.

Studies were excluded if they (1) included non-athletes or participants older than 18 years, (2) did not compare body composition methods, (3) lack relevant body composition outcomes (%FM, FFM, or related parameters), and (4) were case reports, case series, commentaries, abstracts, or conference proceedings. Moreover, AAs were defined as individuals engaged in organized sport activities, competitions and belonging to an athletic community. This definition was chosen to ensure inclusivity across different levels of sports participation, recognizing that body composition assessments are relevant to both competitive and recreational athletes.

### Data extraction

All references were managed using Mendeley, and duplicates were removed. Two independent reviewers screened titles and abstracts, followed by full-text assessments for final inclusion. Discrepancies were resolved through an independent third researcher.

Data were systematically extracted using a structured form, including publication details (title, authors, and year), study characteristics (design and inclusion/exclusion criteria), participant information (sample size, age, sex distribution, and body mass index [BMI] statistics), measurement tools (devices used for DXA, BIA, and other methods; BIA equations applied), results (correlations, limits of agreement, and mean%FM comparisons), and conclusions (key findings and study limitations), missing data were marked as unavailable in the extraction form.

The quality of the studies included in this review was assessed using the NIH Quality Assessment Tool for Observational Cohort and Cross-Sectional Studies. 21​ Each study was evaluated based on 14 key questions, with responses categorized as “yes,” “no,” or “not applicable.” The studies were classified as high quality (11–14 “yes” responses), good quality (7–10 “yes” responses), or poor quality (0–6 “yes” responses). A study’s quality rating was determined by the percentage of “yes” answers, with high-quality studies being considered methodologically robust, good-quality studies having a moderate risk of bias, and poor-quality studies being deemed high risk of bias. Disagreements in ratings were resolved through discussion between the reviewers (for a detailed explanation of the quality assessment tool, see Supplementary Appendix A, available in the online version only).

## Results

**Fig. 2:**
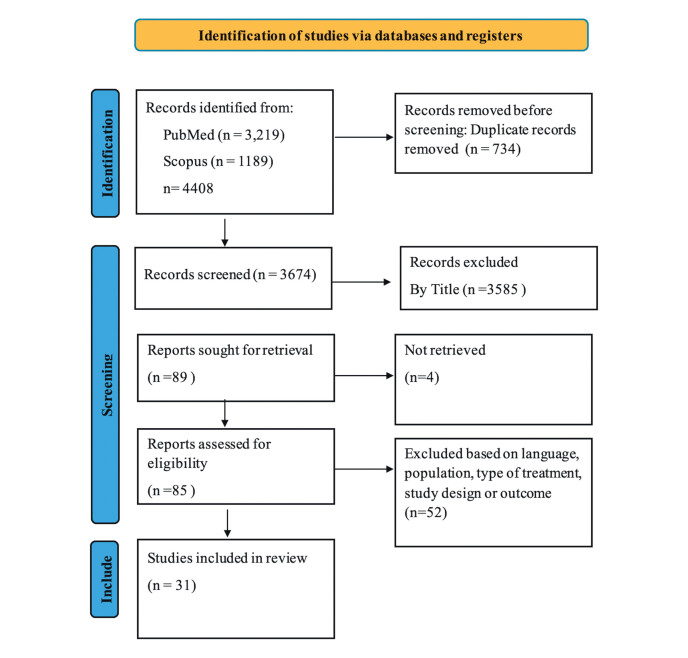
A flow diagram showing the process used to select the study.

The systematic review initially identified 4,408 records. After removing 734 duplicates, 3,674 records underwent title and abstract screening, leading to the exclusion of 3,585 studies. Of the 89 full-text articles retrieved, 4 were excluded due to missing information on inclusion criteria and participants’ characteristics, leaving 85 studies for eligibility assessment. Fifty-two studies were excluded based on language, population criteria, intervention type, study design, or outcome measures, resulting in 31 studies included in the final analysis ( Fig. 2 ). These studies were categorized as follows:

Five studies compared methods without a reference standard, 22​ 23​ 24​ 25​ ​26​
Eleven studies used DXA to validate BIA and/or SF methods, 27​ 28​ 29​ 30​ 31​ 32​ 33​ 34​ 35​ 36​ ​37​
Eight studies used UW as a reference to validate BIA, DXA, SF, and ultra, 38​ 39​ 40​ 41​ 42​ 43​ 44​ ​45​
Three studies involved ADP validation against BIA, DXA, or SF equations, 46​ 47​ ​9​
Two studies compared ADP with four- and five-compartment models, 48​ ​14​
One study validated DXA against a six-compartment model, 15​
One study employed deuterium dilution to validate BIA, DXA, and three prediction equations. 49​

Based on the NIH Quality Assessment Tool for Observational Cohort and Cross-Sectional Studies ( Table 1 ), the studies included in this review were classified into three categories: high, good, and poor quality. Only one study was classified as high quality, with a score of 11 “yes” responses, indicating strong methodological design, robust validation processes, and reliable statistical analysis. Most of the studies (19 studies) were rated as good quality, with scores ranging from 7 to 10 “yes” responses, demonstrating generally sound methodology with some minor limitations. Eight studies were classified as poor quality, scoring fewer than 6 “yes” responses. These studies exhibited significant weaknesses in their methodology, including potential biases in sample size, inconsistent reference methods, missing data, and insufficient statistical analysis.

**Table:** **Table 1** Analysis of methodological quality according to the NIH quality assessment tool for observational cohort and cross-sectional studies

Authors and year	Yes ( *n* )	No ( *n* )	Not given	Quality rating
Eliakim et al. (2000)	7	6	1	Good
Gerasimidis et al. (2014)	8	4	2	Good
de Oliveira-Junior et al. (2016)	9	3	2	Good
Fonseca-Junior et al. (2016)	8	4	2	Good
Leão et al. (2017)	7	2	5	Good
Koury et al. (2018)	7	1	6	Good
Munguia-Izquierdo et al. (2018)	7	1	6	Good
Munguia-Izquierdo et al. (2019)	9	3	2	Good
Núñez et al. (2020)	11	1	2	High
Utczás et al. (2020)	9	2	3	Good
Ramos et al. (2022)	6	6	2	Poor
Housh et al. (2000)	8	1	5	Good
Utter et al. (2005)	9	0	5	Good
Clark et al. (2007)	8	1	5	Good
Utter et al. (2007)	7	1	6	Good
Moon et al. (2008)	5	1	8	Poor
Utter et al. (2009)	6	0	8	Poor
Aerenhouts et al. (2015)	9	1	4	Good
Küçükkubaş et al. (2019)	4	3	7	Poor
Portal et al. (2010)	7	2	5	Good
Ferri-Morales et al. (2018)	7	0	7	Good
Devrim-Lanpir et al. (2021)	8	0	6	Good
Sardinha et al. (2003)	7	0	6	Good
Silva et al., et al. (2003)	4	0	8	Poor
Fügedi et al. (2023)	7	0	7	Good
Silva et al. (2024)	7	0	7	Good
Quiterio et al. (2009)	5	5	4	Poor
Tuuri et al. (2001)	7	3	4	Good
Hetzler et al. (2006)	8	2	4	Good
Berges et al. (2017)	5	6	3	Poor
Liccardo et al. (2021)	6	6	2	Poor

Several studies have compared body fat percentage (%BF) estimation methods without using a reference standard. In a sample of 31 adolescent swimmers (15.1±1.8 y), minimal average differences (~0.9%) in body fat estimates were observed between hydrodensitometry, SF, and DXA, though the individual variation was greater for DXA. 22​ In 208 high school wrestlers (13–18 y), BIA and SF produced significantly different minimum wrestling weight estimates. 23​ Among 92 football players (13.4±0.6 y),%BF comparisons across DXA, ADP, BIA, and SF revealed heteroscedasticity and varying errors. 24​ A study of 20 basketball players (14.95±0.69 y) found high correlations between SF and ultra, despite site-specific differences. 25​ Finally, in 142 elite AAs (11.72±2.33 y), BIA overestimated muscle mass and underestimated%BF compared to anthropometric methods. 26​

Among the studies that considered DXA as the reference method, various SF and BIA techniques were evaluated, with agreement levels differing depending on population, device, and prediction equations. 27​ 28​ 29​ 30​ 31​ 32​ 33​ 34​ 35​ 36​ ​37​ Detailed characteristics of these studies are summarized in Table 2 .

**Table:** **Table 2** Descriptive characteristics of the included studies with DXA as a reference

Authors	Participants	Age (y)	Outcomes	Technology and sampling frequency	Result	Agreement and key findings
Eliakim et al. (2000)	59 female ballet dancers	15.5±0.1	%FM	SF (four sites, Siri’s equation), BIA (RJL system, model 101(50 kHz)	SF *r* =0.80* BIA *r* =0.63*	SF showed closer agreement with DXA
Gerasimidis et al. (2014)	37 (18 females and 19 males) mixed sports	Female: 14.2±1.8 male: 14.4±2.0	%FM FFM (kg)	Tanita -TBF-300 (two equations)	FFM female: *r* =− 0.71* FFM male: *r* =− 0.58*%FM: not reported	Variable agreement; Tanita overestimated%FM in females
de Oliveira-Junior et al. (2017)	43 male soccer	13.3±0.7	%FM FFM (kg)	SF (Slaughter, Lohman); BIA (RJL 101, Houtkooper)	%FM: *r* =0.34–0.98*; FFM: *r* =0.95–0.99*	SF outperformed BIA: Slaughter and Lohman recommended
Leão et al. (2017)	13 male football players	15.8±0.4	%FM	BIA: Tanita BC-418	*r* =0.33*	%FM underestimated by 2.21%
Fonseca-Junior et al. (2017)	51 (24 females and 27 males) Pentathlon	Females: 14.2±2.5 males: 15.1±1.5	%FM	SF (six equations)	females: Md=− 2.03; 2 SD=8.44 males: Md=0.98; 2 SD=7.30	Durnin and Rahaman and Durnin and Womersley equations recommended
Koury et al. (2018)	368 (151 females and 167 males)	Females: 12.8±1.09 males: 12.6±1.02	FM (kg) FFM (kg)	BIA: three equations (Deuremberg, Horlick, and Pietrobelli)	Horlick: males, Rc=0.91, females: 0.87; others<0.12; R ^2^ : males: 0.92, females: 0.84; no statistics reported for% FM	Horlick had best agreement; others showed no agreement; high variability.
Munguia-Izquierdo et al. (2018)	44 male soccer	17.1±0.5	%FM	BIA (Tanita BC-418, InBody 770); SF (11 equations)	SF: *r* =0.51–0.76*; BIA: *r* =− 0.44 to−0.58*	SF performed better than BIA
Munguia-Izquierdo et al. (2019)	00341 male soccer	17.1±0.6	FFM (kg)	BIA (Tanita BC-418, InBody 770); SF (11 equations)	*r* =0.94–0.97* (all methods)	Lower bias in SF; Durnin Womersley, Sarría, and Slaughter recommended
Núñez et al. (2020)	40 male soccer	Pre- season: 16.67±0.5 mid- season; 17.07±0.5	FFM (kg)	BIA (Tanita BC-418, InBody 770); SF (12 equations)	*r* =0.70–0.89*	Minimal bias; multiple SF equations recommended
Utczás et al. (2020)	738 males (soccer, basketball, and handball players)	15.8±1.4	%FM LBM	BIA: InBody 720 (multi-frequency)	Handball:%FM: 8.3±2.4; LBM:−5.0±2.1 kg* Basketball:%FM: 8.8±2.3; LBM:−5.3±1.8 kg* Soccer:%FM: 6.4±2.2; LBM:−3.1±1.4 kg	BIA less accurate in handball/basketball
Ramos et al. (2020)	70>6-month sport participation	11–16	FFM (kg)	Biodynamics-450 (50 kHz), four equations	All equations showed a significant correlation*; Koury MAPE=4.23%, LOA=+ 4.0/−2.6 kg	Koury equation showed the best agreement

Abbreviations: BIA, bioelectrical impedance analysis; DXA, dual-energy X-ray absorptiometry;%FM, fat mass percentage; FFM, fat-free mass; LBM, lean body mass; LOA, limits of agreement; MAPE, mean absolute percentage error; Md, mean difference; *r* , Pearson’s correlation coefficient; Rc, Lin’s concordance correlation coefficient; RMSE, root mean squared error; SD, standard deviation; SEE, standard error of the estimate; SF, skinfold; TBW, total body water.

*Significant differences between methods.

When UW was used as the reference, SF and BIA methods showed variable agreement depending on the population, protocol and prediction equations 38​ 39​ 40​ 41​ 42​ 43​ 44​ ​45​ (see Table 3 ).

**Table:** **Table 3** Descriptive characteristics of the included studies which considered hydrostatic weighing (HW)/underwater weighing (UW) as the reference

Authors	Participants	Age (y)	Outcomes	Technology and sampling frequency	Result	Agreement and key findings
Housh et al. (2000)	137 male Wrestlers	11.3±1.6	BD	16 SF modified equations	Seven equations: *r* =0.62–0.79; SEE=0.0108–0.0139 g/cm ^3^ ; TE=0.0110–0.0152 g/cm ^3^	Moderate agreement across equations
Utter et al. (2004)	129 male Wrestlers	15.5±1.3	FFM	Tanita TBF-300 WA (50 kHz), SF (Brozek eq.)	BIA: 56.9±8.4 kg, *r* =0.93; SK: 56.1±8.9 kg, *r* =0.98; UW: 56.2±9.9 kg; B–A plots: BIA *r* =− 0.39*, SK *r* =− 0.44*	SF preferred due to higher precision in estimating FFM
Clark et al. (2007)	94 wrestlers	16.1±1.2	MWW (based on%FM)	DXA, SF (Brozek equation)	DXA=60.6±9.0 kg, UW=59.8±9.0 kg, *r* =0.98; Lohman SF: 60.1±8.1 kg, *r* =0.97	No systematic bias; DXA is reliable for MWW
Utter et al. (2007)	70 male wrestlers	15.5±1.5	FFM	SF (Brozek eq.), BX-2,000 A-mode ULTRA (2.5 MHz)	ULTRA: 57.2±9.7 kg *r* =0.97 UW: 57.0±9.89 kg; SK: 54.8±8.8* *r* =0.96, B–A plots; ULTRA: *r* =− 0.07, SK: *r* =− 0.38*	ULTRA provides comparable FFM estimates to UW in hydrated adolescents
Moon et al. (2008)	30 males	15.8±1.0	% FM	Tanita BF860 W NIR Futrex 5,000 BOD POD SF (a, b, and c equations based on Jackson and Pollock)	BIA: *r* =0.80, SEE=4.7, TE=6.5*; BP: *r* =0.90, SEE=3.3, TE=3.8*; NIR: *r* =0.86, SEE=3.9, TE=10.4*; SF: *r* =0.91–0.96, SEE=2.3–3.3, TE=3.2–3.5	SF performed best vs. UW; BP did not yield the lowest TE
Utter et al. (2009)	72 wrestlers	15.3±1.4	FFM (kg)	SF (Lohman eq.), InBody 520 (5, 50, 500 kHz)	MFBIA: 57.2 kg, SK: 56.4 kg, UW: 57.0 kg; *r* =0.96–0.97; B–A plots: MFBIA *r* =− 0.22, SK *r* =− 0.47	MFBIA estimates align closely with UW in hydrated youth
Aerenhouts et al. (2015)	N/A	14.8±1.5 females and 14.7±1.9 males	%FM in six time points over 27 mo	Tanita TBF-410 (Slaughter eq.), UW (Siri eq.)	females: BIA *r* =0.30–0.77, SF *r* =0.38–0.77; males: BIA *r* =− 0.04–0.51, SF *r* =0.32–0.64	Low to moderate correlations, especially low agreement with BIA in males
Küçükkubaş et al. (2019)	61 males (basketball, ski, swimming, and handball)	15.90±0.79	Tanita TBF-401 A (50 kHz), Biodynamic 310 (50 kHz), AVIS 333 PLUS (5, 50, 250 kHz)	%FM LBM (kg)	Tanita:%FM *r* =0.66*, LBM *r* =0.96*; Biodynamics:%FM *r* =0.62*, LBM *r* =0.95*; AVIS:%FM *r* =0.63*, LBM *r* =0.93*	Tanita underestimated%BF vs. HW; biodynamics and AVIS overestimated%FM; significant differences between methods

Abbreviations: B–A, Bland–Altman plots; BD, body density; BIA, bioelectrical impedance analysis; BP, bod pod; DXA, dual-energy X-ray absorptiometry;%FM, fat mass percentage; FFM, fat-free mass; HW/UW, hydrostatic/underwater weighing; LBM, lean body mass; MWW, minimum wrestling weight; NIR, near infrared; *r* , Pearson’s correlation coefficient; SEE, standard error of the estimate; SF, skinfold thickness; TE, total error.

*Statistically significant differences between methods.

ADP-based studies included three investigations: one in 29 adolescent volleyball players (16.1±1.3 y) showing strong correlations between SF and BIA ( *r* =0.83) but weak correlation with BMI percentiles ( *r* =0.45); 46​ another in 104 male AAs (13.2±1.0 y) comparing ADP with DXA and BIA, concluding that DXA had superior agreement with ADP ( *r* =0.84 vs. *r* =0.60 for BIA); 47​ and a study in Olympic wrestlers validating SF equations against ADP, recommending sex-specific equations for accurate%BF estimation. 9​

Further studies evaluated ADP accuracy using Lohman’s and Siri’s equations in 51 male AAs (15.5±1.2 y), finding Siri’s equation overestimated%BF compared to Lohman’s and the four-compartment model. 48​ The agreement between ADP and the five-compartment model was superior to DXA, with DXA overestimating%BF in adolescent girls. 14​ The accuracy of the DXA-derived total body protein (TBPro) against a six-compartment model showed that assumed hydration fractions improved accuracy. 15​

Quiterio et al. 49​ assessed TBW estimation in 118 AAs (15.2±1.5 y) using ADP, DXA, and deuterium dilution. The highest accuracy was observed in Lohman’s hydration constants ( *r* ^2^ =0.94 for girls and *r* ^2^ =0.92 for boys), while anthropometric equations showed significant deviations.

Across all included studies ( *n* =31), a total of 1,475 male athletes were reported, whereas only 252 female athletes were identified. Additionally, five studies did not specify the sex of participants. Notably, three of these five studies focused on wrestling and soccer—sports typically dominated by male athletes—suggesting a high probability that these samples were also male-dominated.

## Discussion

The findings reveal substantial variability in accuracy across different techniques, underscoring the importance of selecting context-specific reference methods.

Many studies lacked a consistent reference standard, assessing interchangeability rather than accuracy. 22​ 24​​ ​25​ While BIA and SF methods are practical and widely accessible, they showed inconsistent accuracy, often underestimating%FM and overestimating FFM, especially in athletes with atypical body compositions. 23​ ​26​ SF equations require sport-specific adjustments due to site dependent variability. 50​ 51​ ​52​ However, BIA’s reliance on generalized equations introduces errors, further influenced by age- and sex-related differences. 53​ ​54​

DXA is the most frequently used reference method in the literature. However, its accuracy is affected by hydration status, tissue density variations, and sex-specific factors—particularly in female AAs. 14​ Studies comparing BIA with DXA reveal mixed results, with discrepancies driven by the maturity level, and the equations used. 27​ 32​​ ​36​ Certain SF equations, such as those developed by Slaughter et al. 51​ and Durnin and Womersley, 55​ tended to align more closely with DXA in AAs. 31​ ​34​ However, variations in fat distribution and hydration still posed challenges. Despite its popularity, DXA validation against multi-compartment models in youth remains limited, warranting caution when interpreting DXA-based results. 54​ 56​​ ​57​ For instance, Silva et al. 14​ found that DXA overestimated%BF compared to ADP, particularly in females, likely due to software limitations. 56​ 57​ ​58​

UW shows limited accuracy due to assumptions about tissue density and lung volume variability. 58​ ​59​ Although several studies considered underwater weighing as a reference, its validity in AAs remains uncertain, as none of these studies compared it against multi-compartment models. This limits confidence in its use as a criterion method in this population. ADP, exemplified by the BOD POD, provides a practical alternative to UW by estimating body density via air displacement. Studies in AAs report strong agreement between ADP, DXA, and SF methods, 52​ ​58​ though%BF estimates vary due to density conversion assumptions. 52​ ADP tends to overestimate%BF relative to UW and is influenced by clothing, room temperature, and extreme BMI values. 60​ Additionally, variability in FFM density, especially in AAs and female athletes, can lead to inaccuracies. Nonetheless, comparisons with four-compartment models suggest that integrating ADP into multi-compartment methods may enhance precision. 14​ Deuterium dilution has shown strong agreement with multi-compartment models in adults, confirming its validity for TBW estimation. 61​ However, only one study in this review used it in adolescents, without comparison to multi-compartment models. 49​ Therefore, its accuracy in this population remains unclear.

Multi-compartment models (e.g., 4C, 5C, and 6C) provide the most accurate body composition assessments by accounting for fat, water, proteins, bone minerals, and other components. 56​ They address the limitations of simpler two- and three-compartment methods, which assume a constant FFM density and do not distinguish between bone and soft tissue minerals. 54​ ​59​ Although evidence in AAs is limited, findings from adult populations suggest that increasing compartment details—such as including soft tissue mineral (5C) or glycogen (6C)—can improve accuracy. These additions help account for hydration and tissue variability more effectively than simpler models. 59​ ​61​ These models rely on specialized methods for each component, such as DXA for bone mineral density measurements, deuterium dilution for total body water, and UW for body volume. This targeted approach reduces assumptions and improves precision. However, these models are resource-intensive and not feasible for routine use in sports. 20​ Moreover, no standardized protocols or algorithms currently exist for applying or combining these methods in AAs, highlighting the need for further research to establish practical, age-appropriate assessment strategies.

Various factors such as age, maturation, sex, and athletic discipline must be considered when choosing measurement methods. Using multiple methods is recommended when assessing body compositions in AAs especially when targeting outcomes like%FM or FFM. If only a single method is applied, its limitation should be acknowledged. Additionally, combining body composition data with indicators like energy availability, the risk of relative energy deficiency in sport (REDs) or bone stress injuries can provide a more comprehensive view of athlete health and guide informed decisions in training and nutrition.

Although sex-related differences in measurement accuracy are consistently reported, the evidence base is heavily male-dominated. Across studies, male participants outnumbered females by nearly six to one, limiting the generalizability of findings to female AAs and reinforcing a significant gap in the literature.

A major limitation identified is the inconsistency in study designs and reference methods, which leads to variability and, at times, unreliable results. Differences in measurement protocols, athlete populations, and prediction equations further complicate cross-study comparisons. Variability in sports participation—from general fitness settings to elite competition—as well as differences in anthropometric characteristics, may have influenced the reported accuracy of body composition methods

Based on the NIH Quality Assessment Tool for Observational Cohort and Cross-Sectional Studies ( Table 1 ), most included studies were of good quality, though only one was rated as high quality. Eight studies were classified as poor quality, often due to methodological weaknesses such as small sample sizes, inconsistent reference methods, or limited statistical analysis. Finally, data on AAs remain limited, especially concerning the differentiated effects of sex and age.

## Conclusions

Body composition assessment in AAs lacks a universally accepted gold standard. The precision of laboratory methods varied across studies, suggesting the need for improved standardization and protocol harmonization due to developmental variability and methodological limitations. Simpler field methods offer practicality but require sport- and population-specific adjustments and their accuracy against a valid reference need to be investigated. Multicompartment models provide the highest precision, offering a more comprehensive framework by incorporating multiple body components. However, these models are costly, time-consuming, and impractical for routine sport settings. Comparisons involving multiple methods, particularly when advanced models are included, enhance measurement accuracy.

## Supplementary Material

Supplementary Material Zusatzmaterial

## References

[1] Malina R M Body composition in athletes: Assessment and estimated fatness Clin Sports Med 2007 26 01 37 68 17241914 10.1016/j.csm.2006.11.004

[2] Copic N Dopsaj M Ivanovic J Nesic G Jaric S Body composition and muscle strength predictors of jumping performance: Differences between elite female volleyball competitors and nontrained individuals J Strength Cond Res 2014 28 10 2709 2716 24714534 10.1519/JSC.0000000000000468

[3] Burke L M Loucks A B Broad N Energy and carbohydrate for training and recovery J Sports Sci 2006 24 07 675 685 10.1080/02640410500482602 16766497

[4] Le Gall F Carling C Williams M Reilly T Anthropometric and fitness characteristics of international, professional and amateur male graduate soccer players from an elite youth academy J Sci Med Sport 2010 13 01 90 95 10.1016/j.jsams.2008.07.004 18835220

[5] Malina R M Growth and maturation: Normal variation and the effects of trainingIn: Gisolfi CV, Lamb DR, editorsPerspectives in Exercise Science and Sports MedicineVol. II. Youth, Exercise, and SportBenchmark Press 1989pp.223 265

[6] Malina R M Geithner C A Body composition of young athletes Am J Lifestyle Med 2011 5 03 262 278 10.1177/1559827610392493

[7] Malina R M Rogol A D Cumming S P Coelho e Silva M J Figueiredo A J Biological maturation of youth athletes: Assessment and implications Br J Sports Med 2015 49 13 852 859 10.1136/bjsports-2015-094623 26084525

[8] Grigoletto A Mauro M Toselli S Differences in body composition and maturity status in young male volleyball players of different levels J Funct Morphol Kinesiol 2023 8 04 162 10.3390/jfmk8040162 38132717 PMC10744010

[9] Devrim-Lanpir A Badem E A Işık H Çakar A N Kabak B Akınoğlu B Which body density equations calculate body fat percentage better in Olympic wrestlers? Comparison study with air displacement plethysmography Life 2021 11 07 707 10.3390/life11070707 34357079 PMC8306702

[10] Hawkinson J Timins J Angelo D Shaw M Takata R Harshaw F Technical white paper: Bone densitometry J Am College Radiol 2007 4 05 320 327 10.1016/j.jacr.2007.01.021 17467615

[11] Gibby J T Njeru D K Cvetko S T Heiny E L Creer A R Gibby W A Whole-body computed tomography-based body mass and body fat quantification: A comparison to hydrostatic weighing and air displacement plethysmography J Comput Assisted Tomogr 2017 41 02 302 308 10.1097/RCT.0000000000000516 27753722

[12] Hume P Marfell-Jones M The importance of accurate site location for skinfold measurement J Sports Sci 2008 26 12 1333 1340 10.1080/02640410802165707 18821122

[13] Ackland T R Lohman T G Sundgot-Borgen J Maughan R J Meyer N L Stewart A D Current status of body composition assessment in sport: Review and position statement on behalf of the Ad Hoc Research Working Group on Body Composition Health and Performance, under the auspices of the I.O.C. Medical Commission Sports Med 2012 42 03 227 249 22303996 10.2165/11597140-000000000-00000

[14] Silva A M Minderico C S Teixeira P J Pietrobelli A Sardinha L B Body fat measurement in adolescent athletes: Multicompartment molecular model comparison Eur J Clin Nutr 2006 60 08 955 964 10.1038/sj.ejcn.1602405 16523205

[15] Silva A M Campa F Sardinha L B The usefulness of total body protein mass models for adolescent athletes Front Nutr 2024 11 1.439208E6 10.3389/fnut.2024.1439208 PMC11262245 39040929

[16] Sellés-Pérez S Fernández-Sáez J Férriz-Valero A Esteve-Lanao J Cejuela R Changes in triathletes’ performance and body composition during a specific training period for a Half-Ironman race J Human Kinet 2019 67 185 198 10.2478/hukin-2018-0077 31523317 PMC6714369

[17] Coppini L Z Waitzberg D L Campos AC L Limitations and validation of bioelectrical impedance analysis in morbidly obese patients Curr Opin Clin Nutr Metab Care 2005 8 03 329 332 15809537 10.1097/01.mco.0000165013.54696.64

[18] Loenneke J P Wilson J M Barnes J T Pujol T J Validity of the current NCAA minimum weight protocol: A brief review Ann Nutr Metab 2011 58 03 245 249 10.1159/000330574 21846975

[19] Poureghbali S Engel T 2025 https://www.crd.york.ac.uk/PROSPERO/view/CRD420251027825

[20] Baracos V Caserotti P Earthman C P Fields D Gallagher D Hall K D Advances in the science and application of body composition measurement JPEN, J Parenter Enteral Nutr 2012 36 01 96 107 10.1177/0148607111417448 22235108 PMC4422066

[21] Delavari S Pourahmadi M Barzkar F What quality assessment tool should I use? A practical guide for systematic reviews authors Iran J Med Sci 2023 48 03 229 231 10.30476/IJMS.2023.98401.3038 37791333 PMC10542923

[22] Tuuri G Loftin M Comparison of hydrodensitometry, skinfold thickness, and dual-energy X-ray absorptiometry for body fat estimation in youth swimmers Pediatric Exerc Sci 2001 13 03 238 245 10.1123/pes.13.3.238

[23] Hetzler R K Kimura I F Haines K Labotz M Smith J A comparison of bioelectrical impedance and skinfold measurements in determining minimum wrestling weights in high school wrestlers J Athletic Training 2006 41 01 46 51 PMC1421483 16619094

[24] Lozano Berges G Matute Llorente Á Gómez Bruton A González Agüero A Vicente Rodríguez G Casajús J A Body fat percentage comparisons between four methods in young football players: Are they comparable? Nutr Hosp 2017 34 05 1119 1124 10.20960/nh.760 29130710

[25] Liccardo A Tafuri D Corvino A Body composition analysis in adolescent male athletes: Skinfold versus ultrasound J Human Sport Exerc 2021 16Proc2S59 S67 10.14198/jhse.2021.16.Proc2.08

[26] Fügedi B Szakály Z Suszter L Comparison of the results of bioelectric impedance analysis (BIA) and the anthropometry (Drinkwater-Ross & Parizkova) method in young elite athletes J Phys Educ Sport 2023 23 01 247 254 10.7752/jpes.2023.01030

[27] Eliakim A Ish-Shalom S Giladi A Falk B Constantini N Assessment of body composition in ballet dancers: Correlation among anthropometric measurements, bio-electrical impedance analysis, and dual-energy X-ray absorptiometry Int J Sports Med 2000 21 08 598 601 10.1055/s-2000-8489 11156282

[28] Gerasimidis K Shepherd S Rashid R Edwards C A Ahmed F Group and individual agreement between field and dual X-ray absorptiometry-based body composition techniques in children from standard schools and a sports academy J Acad Nutr Diet 2014 114 01 91 98 10.1016/j.jand.2013.06.353 24021735

[29] Leão C Simões M Silva B Clemente F M Bezerra P Camões M Body composition evaluation issue among young elite football players: DXA assessment Sports 2017 5 01 17 10.3390/sports5010017 29910377 PMC5969018

[30] Fonseca-Junior S J Oliveira A J Loureiro L L Pierucci AP T Validity of skinfold equations, against dual-energy X-ray absorptiometry, in predicting body composition in adolescent pentathletes Pediatric Exerc Sci 2017 29 02 285 293 10.1123/pes.2016-0101 27705535

[31] Oliveira Junior A Casimiro G Donangelo C Farinatti P Massuça L Fragoso I Methodological agreement between body-composition methods in young soccer players stratified by zinc plasma levels Int J Morphol 2016 34 01 49 56 10.4067/S0717-95022016000100008

[32] Koury J C Ribeiro M A Massarani F A Vieira F Marini E Fat-free mass in adolescent athletes: Accuracy of bioimpedance equations and identification of new predictive equations Nutrition 2019 60 59 65 10.1016/j.nut.2018.09.029 30529187

[33] Munguia-Izquierdo D Suarez-Arrones L Di Salvo V Paredes-Hernandez V Alcazar J Ara I Validation of field methods to assess body fat percentage in elite youth soccer players Int J Sports Med 2018 39 05 349 354 10.1055/s-0044-101145 29564845

[34] Munguía-Izquierdo D Suárez-Arrones L Di Salvo V Paredes-Hernández V Ara I Mendez-Villanueva A Estimating fat-free mass in elite youth male soccer players: Cross-validation of different field methods and development of prediction equation J Sports Sci 2019 37 11 1197 1204 10.1080/02640414.2018.1543833 30526374

[35] Núñez F J Munguía-Izquierdo D Suárez-Arrones L Validity of field methods to estimate fat-free mass changes throughout the season in elite youth soccer players Front Physiol 2020 11 16 10.3389/fphys.2020.00016 32116741 PMC7029743

[36] Utczas K Tróznai Z Pálinkás G Kalabiska I Petridis L How length sizes affect body composition estimation in adolescent athletes using bioelectrical impedance J Sports Sci Med 2020 19 03 577 584 32874111 PMC7429426

[37] Ramos I E Coelho G M Lanzillotti H S Marini E Koury J C Fat-free mass using bioelectrical impedance analysis as an alternative to dual-energy X-ray absorptiometry in calculating energy availability in female adolescent athletes Int J Sport Nutr Exerc Metab 2022 32 05 350 358 10.1123/ijsnem.2021-0301 35523421

[38] Housh T J Johnson G O Housh D J Stout J R Eckerson J M Schätzungdichte bei Ringern [Estimation density in wrestlers] J Strength Cond Res 2000 14 04 477 482

[39] Clark R R Sullivan J C Bartok C J Carrel A L DXA provides a valid minimum weight in wrestlers Med Sci Sports Exerc 2007 39 11 2069 2075 10.1249/mss.0b013e31814fb423 17986917

[40] Utter A C Nieman D C Mulford G J Tobin R Schumm S McInnis T Evaluation of leg-to-leg BIA in assessing body composition of high-school wrestlers Med Sci Sports Exerc 2005 37 08 1395 1400 10.1249/01.mss.0000174901.05353.f2 16118588

[41] Utter A C Hager M E Evaluation of ultrasound in assessing body composition of high school wrestlers Med Sci Sports Exerc 2008 40 05 943 949 10.1249/MSS.0b013e318163f29e 18408602

[42] Utter A C Lambeth P G Evaluation of multifrequency bioelectrical impedance analysis in assessing body composition of wrestlers Med Sci Sports Exerc 2010 42 02 361 367 10.1249/MSS.0b013e3181b2e8b4 19927023

[43] Moon J R Tobkin S E Costa P B Smalls M Mieding W K O’Kroy J A Validity of the BOD POD for assessing body composition in athletic high school boys J Strength Cond Res 2008 22 01 263 268 10.1519/JSC.0b013e31815f60ce 18296985

[44] Aerenhouts D Clarys P Taeymans J Van Cauwenberg J Estimating body composition in adolescent sprint athletes: Comparison of different methods in a 3 years longitudinal design PLoS One 2015 10 08 e0136788 10.1371/journal.pone.0136788 26317426 PMC4552792

[45] Küçükkubaş N Hazır Aytar S Acikada C Hazır T Bioelectric impedance analyses for young male athletes: A validation study Isokinetics Exerc Sci 2019 28 01 1 10 10.3233/IES-185209

[46] Portal S Rabinowitz J Adler-Portal D Burstein R P Lahav Y Meckel Y Body fat measurements in elite adolescent volleyball players: Correlation between skinfold thickness, bioelectrical impedance analysis, air-displacement plethysmography, and body mass index percentiles J Pediatric Endocrinol Metab 2010 23 04 395 400 10.1515/jpem.2010.061 20583545

[47] Ferri-Morales A Nascimento-Ferreira M V Vlachopoulos D Ubago-Guisado E Torres-Costoso A De Moraes AC F Agreement between standard body composition methods to estimate percentage of body fat in young male athletes Pediatric Exerc Sci 2018 30 03 402 410 10.1123/pes.2017-017 29543127

[48] Sardinha L B Silva A M Teixeira P J Usefulness of age-adjusted equations to estimate body fat with air displacement plethysmography in male adolescent athletes Acta Diabetol 2003 40(Suppl 1)S63 S67 10.1007/s00592-003-0029-7 14618436

[49] Quiterio A L Silva A M Minderico C S Carnero E A Fields D A Sardinha L B Total body water measurements in adolescent athletes: A comparison of six field methods with deuterium dilution J Strength Cond Res 2009 23 04 1225 1237 10.1519/JSC.0b013e3181a9ec39 19568032

[50] Lohman T G Applicability of body composition techniques and constants for children and youths Exerc Sport Sci Rev 1986 14 325 357 3525188

[51] Slaughter M H Lohman T G Boileau R A Horswill C A Stillman R J Van Loan M D Skinfold equations for estimation of body fatness in children and youth Human Biol 1988 60 05 709 723 3224965

[52] Kasper A M Langan-Evans C Hudson J F Brownlee T E Harper L D Naughton R J Come back skinfolds, all is forgiven: A narrative review of the efficacy of common body composition methods in applied sports practice Nutrients 2021 13 04 1075 10.3390/nu13041075 33806245 PMC8065383

[53] Pietrobelli A Faith M S Allison D B Gallagher D Chiumello G Heymsfield S B Body mass index as a measure of adiposity among children and adolescents: A validation study J Pediatr 1998 132 02 204 210 10.1016/s0022-3476(98)70433-0 9506629

[54] Pietrobelli A Gallagher D Baumgartner R Ross R Heymsfield S B Lean R value for DXA two-component soft-tissue model: Influence of age and tissue or organ type Appl Radiat Isot 1998 49 5/6 743 744 10.1016/s0969-8043(97)00100-0 9569598

[55] Durnin J V Womersley J Body fat assessed from total body density and its estimation from skinfold thickness: Measurements on 481 men and women aged from 16 to 72 years Br J Nutr 1974 32 01 77 97 10.1079/bjn19740060 4843734

[56] Sopher A B Thornton J C Wang J Pierson R N Heymsfield S B Horlick M Measurement of percentage of body fat in 411 children and adolescents: A comparison of dual-energy X-ray absorptiometry with a four-compartment model Pediatrics 2004 113 05 1285 1290 10.1542/peds.113.5.1285 15121943 PMC4418431

[57] Williams J E Wells J C Wilson C M Haroun D Lucas A Fewtrell M S Evaluation of Lunar Prodigy dual-energy X-ray absorptiometry for assessing body composition in healthy persons and patients by comparison with the criterion 4-component model Am J Clin Nutr 2006 83 05 1047 1054 10.1093/ajcn/83.5.1047 16685045

[58] Fields D A Goran M I Body composition techniques and the four-compartment model in children J Appl Physiol 2000 89 02 613 620 10.1152/jappl.2000.89.2.613 10926645

[59] Fuller N J Jebb S A Laskey M A Coward W A Elia M Four-component model for the assessment of body composition in humans: Comparison with alternative methods, and evaluation of the density and hydration of fat-free mass Clin Sci 1992 82 06 687 693 10.1042/cs0820687 1320550

[60] Going S B Hydrodensitometry and air displacement plethysmographyIn: Heymsfield SB, Lohman TG, Wang ZM, Going SJ, editorsHuman body composition2nd ednHuman Kinetics 2005pp.17 33

[61] Wang Z Pi-Sunyer F X Kotler D P Wielopolski L Withers R T Pierson R N Multicomponent methods: Evaluation of new and traditional soft tissue mineral models by in vivo neutron activation analysis Am J Clin Nutr 2002 76 04 968 977 12399267 10.1093/ajcn/76.5.968

